# CK1α overexpression correlates with poor survival in colorectal cancer

**DOI:** 10.1186/s12885-018-4019-0

**Published:** 2018-02-06

**Authors:** Julia Richter, Anna-Laura Kretz, Johannes Lemke, Michael Fauler, Jens-Uwe Werner, Stephan Paschke, Frank Leithäuser, Doris Henne-Bruns, Andreas Hillenbrand, Uwe Knippschild

**Affiliations:** 1 Department of General and Visceral Surgery, Ulm University Hospital, Albert-Einstein-Allee 23, 89081 Ulm, Germany; 2 Ulm University, Institute of General Physiology, Albert-Einstein-Allee 11, 89081 Ulm, Germany; 3 Department of Pathology, Ulm University Hospital, Albert-Einstein-Allee 23, 89081 Ulm, Germany

**Keywords:** Casein kinase 1 alpha, Biomarker, Colorectal cancer (CRC), Survival, Prognosis, Drug target

## Abstract

**Background:**

Colorectal cancer (CRC) is the fourth leading cause of cancer related deaths worldwide and prognosis in advanced tumor stage still remains poor. Since CK1 isoforms have been reported to be deregulated in several tumor entities CK1 has emerged as a novel drug target in cancer therapy. In this study we set out to investigate whether CK1α might have the potential to serve as prognostic marker.

**Methods:**

CK1α RNA and protein expression levels in healthy and tumor tissue of CRC patients were analyzed using quantitative real-time PCR and Western Blot analysis, respectively. Prognostic relevance was investigated by correlating obtained CK1α expression levels with patients’ survival rate generating Kaplan-Meier survival plots.

**Results:**

It could be shown that CK1α is overexpressed in colorectal tumor tissue compared to normal tissue and CK1α overexpression in tumor tissue correlates with poor survival in CRC patients. Results become more significant when only considering patients with high-grade tumors, as well as patients assigned to UICC II and UICC III stage. Furthermore, Cox regression analysis revealed that CK1α is an independent prognostic factor. In addition, tumors expressing decreased levels of the kinase reveal positive effects on overall survival when localized in the right colon compared to those in the left side.

**Conclusion:**

In summary, this study provides evidence for the first time that CK1α RNA levels might serve as prognostic marker for CRC.

**Electronic supplementary material:**

The online version of this article (10.1186/s12885-018-4019-0) contains supplementary material, which is available to authorized users.

## Background

According to the GLOBOCAN statistics an incidence of 1,360,000 estimated cases of colorectal cancer (CRC) globally occurred in 2012, thereby representing the third most common cancer in men (746,000 cases, 10% of total) after lung and prostate cancer and the second in women (614,000 cases, 9.2% of total) following breast cancer. Almost 55% of the CRC cases have been accounted in developed regions with most cases depicted in Australia and New Zealand, followed by Western and Southern Europe [1]. In fact, CRC is the fourth leading cause of cancer related deaths resulting largely from the inefficiency of early detection and resistance to chemotherapy [2]. Older age, lifestyle, dietary, and the gut microbiota influence lifetime risk of CRC (reviewed in [3]). Colorectal carcinogenesis is a process over several years usually arising from benign adenomatous polyps of the colonic mucosa which eventually develop malignancy. CRC can infiltrate into other intestinal layers and might invade lymph or blood vessels, giving rise to metastasis in lymph nodes or distant organs [4]. Despite extensive research in CRC, its pathogenesis is still not fully understood. However, based on Fearon and Vogelstein’s findings, the adenoma-carcinoma sequence describes a multi-step process for CRC development, promoted by specific and well-defined genetic aberrations of the genes APC (adenomatous polyposis coli), KRAS (GTPase *KRas)*, DCC (Deleted in Colorectal Cancer) and TP53 (Tumor protein 53) [5]. However, up to 30% of the CRC cases are described as an inherited variant including hereditary non-polyposis colon cancer (HNPCC/Lynch syndrome) and familial adenomatous polyposis coli (FAP) as the most common forms of hereditary CRC (reviewed in [6, 7]). Approximately 15% of CRC development is based on microsatellite instability (MSI-H) due to either germline mutations in one of the mismatch repair genes MLH1, MSH2, MSH6, and pMSM2 (Lynch Syndrome), or to sporadic mutations in the DNA mismatch repair pathway which predominantly occurs through hypermethylation of the MLH1 promotor region and is often associated with the BRAFV600E mutation (reviewed in [8, 9]).

The standard therapy for CRC is surgical resection combined with adjuvant chemotherapy for patients with high risk stage II or III. Recent improvement of the chemotherapeutic regimes supplemented with biological reagents such as EGFR (epidermal growth factor receptor) antagonists have been shown to significantly improve patients’ overall survival [10]. Despite these advances in systemic therapy, the 5-year-survival of metastatic CRC patients remained a mere 12.5% and the main reason for therapy failure seems to be due to acquired treatment resistance occurring in 90% of the patients [11, 12]. Mechanisms described to circumvent the drug delivery include upregulation, mutations or activation of downstream signaling molecules being involved in oncogenic pathways, as well as pathway bypass mechanisms or increased networking between cancer promoting pathways (reviewed in [13]). With respect to the high mortality rate of advanced CRC identification of new biomarkers for prognosis paving the way for new and possibly individual therapeutic approaches are urgently needed.

So far, the general role of CK1 isoforms in cancer development and progression has been revealed (reviewed in [14, 15]) and several reports indicate a potential role of CK1 in digestive cancer screening (reviewed in [16]). However, the prognostic relevance of CK1α in CRC as well as its potential as a therapeutic target has not yet been addressed in detail. Recently, we reported that low CK1δ expression is associated with increased survival rates in CRC patients, especially in patients with highly differentiated tumors [17]. The aim of this study was to investigate whether CK1α is overexpressed in CRC tissue and whether CK1α RNA levels correlate with the overall survival of CRC patients. Furthermore, we wondered if CK1α expression levels and survival of patients correlate with tumor localization. The results presented in this study indicate that CK1α RNA levels might serve as prognostic biomarker for CRC as increased kinase levels correlate with poor survival.

## Methods

### Human tumor tissue

In summary, 283 patients suffering from CRC, who underwent curative tumor resection between 2003 and 2014 at the Department of General and Visceral Surgery at the University of Ulm, Germany, a Certified Intestinal Cancer Center, were included in the study. Informed consent was obtained prior to surgery. Exclusion criteria were age < 18 years, pregnancy, or viral infections like HIV or hepatitis B infection. Moreover, in cases without sufficient amounts of tumor and healthy tissue for processing, patients were not included in the analyses. None of the patients received neoadjuvant treatment prior to the surgery. Tissue samples were collected during operation and specimens were subjected to routine pathological analysis. Thereafter, pathologists provided tumor tissue as well as normal large intestinal mucosa, with at least 3 cm distance to the tumor interface for our tissue data bank.

Retrospectively, clinical data were reviewed based on the departmental records including medical history and on histopathological results from contributing pathologists. The following variables were considered: gender, histologic differentiation, T classification, lymph node invasion, distant metastasis, tumor stage (according to the Union for International Cancer Control, UICC [18, 19]), tumor localization (proximal (right) and distal (left) to the splenic flexure), age, disease free survival and overall survival. The study was performed with the permission of the independent local ethics committee of the University of Ulm (approvals 112/2003, 268/2008, and 235/2015).

### Quantification of gene expression by quantitative real-time PCR

RNeasy Mini Kit (Qiagen, Hilden, Germany) was used to isolate total RNA from frozen tumor tissue sections of CRC patients and 1 μg of total RNA was transcribed into cDNA using the AffinityScript cDNA Synthesis Kit (Agilent Technologies, Santa Clara, USA). PCR amplification of the β-actin housekeeping gene verified RNA integrity and fidelity of cDNA synthesis by using the exon/exon spanning β-actin primer pair β-actin_for (5’-GGC ATC CTC ACC CTG AAG TA-3′) and β-actin_rev (5-‘GTC AGG CAG CTC GTA GCT CT-3’), while the β-actin intron/exon spanning primer pair β-actin_I (5′-cga gca gga gat ggc cac tgC-3′) and β-actin_E (5′-GTG AGC TCT CTG GGT GCT GGG-3′) was used to detect contaminations with genomic DNA. Quantitative gene expression of CK1α (EC 2.7.11.1) was analyzed using the LC480 cycler (Roche, Mannheim, Germany), QuantiFast SYBR Green PCR Kit (Qiagen, Hilden, Germany), and validated QuantiTect Primer Assay (Hs_CSNK1A1_1_SG QT00999138, Qiagen, Hilden, Germany) guaranteeing highly specific and sensitive results in RT-PCR. An interval of Ct values from 18 to 35 was accepted. In order to exclude primer dimers and to guarantee reaction specificity, melting points were analyzed after amplification.

Primers for HPRT (Hs_HPRT1_1_SG QT00059066) were used as endogenous control for all target genes. All experiments were done in duplicates. Results are shown as ∆Ct values.

### Western blot analysis

To detect CK1α protein levels in normal and tumor tissue of CRC patients, samples were lysed in NP40 lysis buffer containing 50 mM Tris-HCl, pH 8.0, 120 mM NaCl, 10% glycerol, 0.5% NP-40, 1 mM EGTA, 5 mM DTT, 200 mM PMSF, 1 mM benzamidine, and 25 μg/ml aprotinin. After clearing of lysates by centrifugation at 15,000 g for 30 min, protein concentrations were determined using the ‘BCA Protein Assay Kit’ (Pierce Biotechnology, Rockford, USA). In each case 20 μg protein lysate were separated on SDS gels, transferred to nitrocellulose membranes (Amersham™ Protran®, GE Healthcare, Munich, Germany) and probed with anti CK1α (C19, 1:1000) (SantaCruz Biotechnology, Heidelberg, Germany) or anti GAPDH (Glyceraldehyde-3-phosphate dehydrogenase; 1:5,000) (HighTest Ltd., Turku, Finland) specific primary antibodies overnight. Detection was performed by using horseradish peroxidase-conjugated anti-goat (1:10,000) (abcam, ab6741, Cambrigde, UK) or anti-mouse IgG (1:10,000) (GE Healthcare, UK) as secondary antibodies, followed by chemiluminescence detection on films. Densitometric analysis was performed by the use of ImageJ software. After background subtraction, density was calculated in comparison to the loading control. Difference between relative density of tumor tissue and normal tissue was calculated and the entirety of values separated by the median in protein expression of tumor tissue lower/equal and higher, respectively, to normal tissue.

### Statistical analysis

SPSS 24 (SPSS Inc., USA) was used performing exploratory data analysis for investigation of the obtained data (raw data is supplied as Additional file 1). Kaplan-Meier estimation was created for statistical analysis of overall survival and significance was tested using log-rank test. Independent prognostic factors were identified by fitting Cox proportional hazards regression models. Inclusion of covariates into the model was based on the forward stepwise likelihood-ratio procedure of the COXREG command in SPSS. The variable with the smallest *p*-value was included into the model of the next step. The inclusion procedure was stopped if no variable had a *p*-value smaller than 0.05. Covariates under consideration were UICC score, patient gender and age, risk factors smoking and alcohol consumption, categorical indicator variables for post-surgery chemotherapy, tumor location (left, right colon or transverse colon) and tumor recurrence. The CK1α expression level was encoded as an indicator variable with a value of 1 if the expression value was above a threshold. The threshold was set to 2.6, a value at which there was a significant difference in the survival rate between the separated groups in the study population (see Additional file 2). Group comparisons were performed by applying Wilcoxon test. *p*-values < 0.05 were considered statistically significant (a = 0.05). No correction for multiple testing was done.

## Results

### Study population

The clinical and histopathological parameters of the CRC patient cohort are listed in Table 1. Tumor tissue of 283 CRC patients (160 male, 123 female), with a median age of 70.13 years (range 29.81–89.68 years) was analyzed in the study. The classification by tumor grade results in dissimilar groups of 209 patients with low-grade (Grade 1, Grade 2; 73.9%) and 71 patients with high-grade (Grade 3, Grade 4; 25.1%) tumors. Among these cases, 74 patients had T1 or T2 status (26.1%), and 205 patients exhibited T3 or T4 status (72.5%). In 135 patients lymph node metastases were evident (47.7%) but only 71 patients had distant metastasis (25.1%). In summary, 62 patients were diagnosed at stage I according to the UICC classification (21.9%), 68 patients at stage II (24.0%), 78 patients at stage III (27.6%), and 71 patients in stage IV (25.1%). Furthermore, distinction into right- and left-sided colon cancer splits groups into 119 patients with tumors localized in right colon (42.1%) and 160 patients with tumors grown in left colon (56.5%). The median overall survival was 28.19 months ranging from 0.13 to 141.38 months. The 5-year survival was 52.9%.

**Table 1:** CRC patients’ characteristics

Variable		*N* = 283	%
Gender	Male	160	56.5
	Female	123	43.5
Differentiation	Grade 1	18	6.4
	Grade 2	191	67.5
	Grade 3	64	22.6
	Grade 4	7	2.5
	n.d.	3	1.1
T classification	T1	19	6.7
	T2	55	19.4
	T3	159	56.2
	T4	46	16.3
	cis	4	1.4
Lymph node invasion	No	148	52.3
	Yes	135	47.7
Distant metastasis	No	212	74.9
	Yes	71	25.1
Stage (UICC)	0	4	1.4
	I	62	21.9
	II	68	24.0
	III	78	27.6
	IV	71	25.1
Tumor localization	Left colon	160	56.5
	Right colon	119	42.1
	n.d.	4	1.4
5-year survival			52.9
Age (years)	Median	70.13	
	Range	29.81–89.68	
Overall survival (months)	Median	28.16	
	Range	0.13–141.38	

*Abbreviations: n.d* not determined, *cis* carcinoma in situ

### CK1α is overexpressed in colorectal tumor tissue

**Fig. 1:**
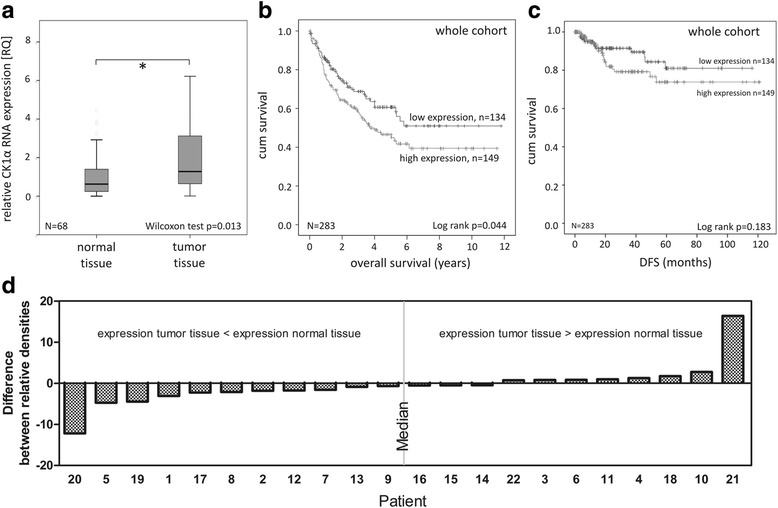
Investigation of CK1α RNA expression as a prognostic marker in CRC. **a** Box plot representing group comparison of relative CK1α RNA expression in normal and tumor tissue of CRC patients. **b** Kaplan-Meier plot displaying the overall survival of whole cohort, divided according to relative CK1α RNA expression in tumor tissue. *p < 0.05. CK1α RNA expression in colorectal tumor tissue of whole cohort was relatively quantified by qPCR using specific primers. HPRT gene served as reference gene. Graphs were created using IBM SPSS Statistics 20. **c** Analysis of disease-free survival (DFS) of CRC patients. Kaplan-Meier plot mapping DSF of whole cohort according to relative CK1α RNA expression in tumor tissue. *p < 0.05. CK1α RNA expression in colorectal tumor tissue of whole cohort was relatively quantified by qPCR using specific primers. HPRT gene served as reference gene. Graphs were created using IBM SPSS Statistics 20. **d** Western Blot Analysis of CK1α protein expression in colorectal normal and tumor tissue. CRC patients of the described cohort were randomly chosen and their normal and tumor tissue subjected to Western Blot analysis. Chemiluminescent blots were visualized on films and densitometry of bands analyzed using ImageJ software. Difference between relative density of tumor tissue and normal tissue was calculated and the entirety of values separated by the median in protein expression of tumor tissue lower/equal and higher/equal, respectively, to normal tissue. Data was drawn as a graph using GraphPad Prism 6 software

Evidence has been provided in the last decade emphasizing the crucial role of CK1 isoforms in cancer development in different tumor entities. For instance, microarray data-base analyses from tumor cell lines and tissues indicate that CK1α is overexpressed on RNA level in many tumor types, including CRC (CellMiner™ database [20]). Initially, to investigate if CK1α could be a potential biomarker in colorectal cancer expression levels in healthy and carcinogenic colorectal tissue were compared in a randomly chosen subset of 68 CRC patients performing quantitative real-time PCR. Results revealed significantly increased CK1α RNA expression levels in tumor tissue compared to healthy samples (*p* = 0.013; Fig. 1A). Furthermore, to confirm a correlation between CK1α RNA and protein expression levels, several patients of the cohort were chosen on a random basis for the detection of CK1α protein levels in Western Blot analysis (Fig. 1D). Indeed, we found equal or lower CK1α expression levels in tumors compared to normal tissue in patients with low CK1α RNA levels (RQ < 2.6; Fig. 1B) as well as strong CK1α immunoreactivity in patients with high CK1α RNA levels (RQ > =2.6; Fig. 1B).

### CK1α is a negative prognostic marker in CRC

In order to investigate CK1α transcript expression as a prognostic factor of CRC, Kaplan-Meier survival plots were generated by correlating low (RQ < 2.6) and high (RQ > = 2.6) CK1α RNA expression levels with 283 patients’ overall survival rates. This threshold separates the study population into two groups with significant different hazard rates. There is no linear dose-response relationship between CK1α expression and survival rate (data not shown) but a tendency for an increasing hazard with expression values up to 1.6 (Additional file 3). Firstly, CK1α RNA expression levels in tumors of the whole patient cohort were investigated, relinquishing significantly increased survival rates of patients suffering from tumors expressing low levels of CK1α (*p* = 0.044, Fig. 1B). After 10-year follow up disease free survival (DFS) was better in CRC patients with low CK1α levels (Fig. 1C).

**Fig. 2:**
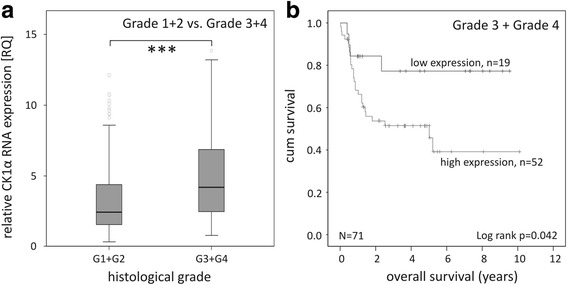
Tumor differentiation-dependent investigation of CK1α RNA expression. **a** Box plot representing group comparison of relative CK1α RNA expression in low-grade and high-grade CRC patients. **b** Kaplan-Meier plot displaying the overall survival of Grade 3 and Grade 4 tumors of CRC patients, divided according to relative CK1α RNA expression. *** *p* < 0.001. CK1α RNA expression in colorectal tumor tissue of all grades was relatively quantified by qPCR using specific primers. HPRT gene served as reference gene. Graphs were created using IBM SPSS Statistics 20

Next, possible gender-specific differences were analyzed by correlating the survival rates for males and females to the expression levels of the kinase. Neither in females, nor in males results revealed a significant correlation of CK1α RNA expression with overall survival rates (*p* = 0.185 and *p* = 0.128, respectively; Additional file 4). Furthermore, within the subgroup of gender no significant differences in median CK1α expression could be observed (*p* = 0.364; Table 2). Differentiating the cohort into two subgroups of low (Grade 1, Grade 2)- and high-grade (Grade 3, Grade 4) classified tumors revealed significantly increased CK1α expression levels in high-grade tumors (*p* < 0.001; Fig. 2A). In addition, as shown in Fig. 2B, patients within the high-grade tumor-subgroup had significantly prolonged survival rates when CK1α expression is decreased (*p* = 0.042). In contrast, in patients with low-grade neoplasms overall survival did not correlate with the level of CK1α RNA expression (*p* = 0.393; Additional file 5).

**Table 2:** Correlation of CK1α RNA expression levels with clinical parameters

	Total N	low CK1α expression [RQ < 2.6] N (median RQ)	high CK1α expression [RQ > =2.6] N (median RQ)	median CK1α expression RQ (min-max)	*p* value
Overall Survival					
Dead	120	46 (1.69)	74 (4.70)	3.23 (0.49–59.92)	0.036*
Alive	163	88 (1.61)	75 (5.06)	2.46 (0.34–67.42)	
Gender					
Male	160	71 (1.66)	89 (4.87)	2.89 (0.49–67.42)	0.364
Female	123	63 (1.63)	60 (4.85)	2.54 (0.34–59.92)	
Differentiation					
Low-grade (G1, G2)	209	115 (1.65)	94 (4.58)	2.43 (0.34–67.42)	< 0.001**
High-grade (G3, G4)	71	19 (1.67)	52 (5.38)	4.17 (0.77–53.30)	
n.d.	3	–	3 (8.11)	8.11 (3.24–8.31)	
Stage (UICC)					
0	4	1 (1.71)	3 (8.31)	6.38 (1.71–10.09)	
I	62	27 (1.69)	35 (4.84)	2.78 (0.64–67.42)	0.611
II, III	146	74 (1.63)	72 (4.76)	2.50 (0.34–59.92)	
IV	71	32 (1.61)	39 (5.07)	2.98 (0.53–53.30)	
Localization					
Left colon	160	76 (1.70)	84 (4.90)	2.71 (0.34–67.42)	0.523
Right colon	119	57 (1.63)	62 (4.70)	2.84 (0.53–15.58)	
n.d.	4	1 (2.39)	3 (7.03)	5.21 (2.39–9.72)	
Total	283	134 (1.66)	149 (4.87)	2.76 (0.34–67.42)	

Clinical parameters referring to CK1α RNA expression levels, calculated as relative quantification (RQ) by qPCR using HPRT as reference gene

Statistical analysis was performed using median test. *p < 0.05; ***p < 0.001

*Abbreviations: G* Grade, *n.d* not determined

**Fig. 3:**
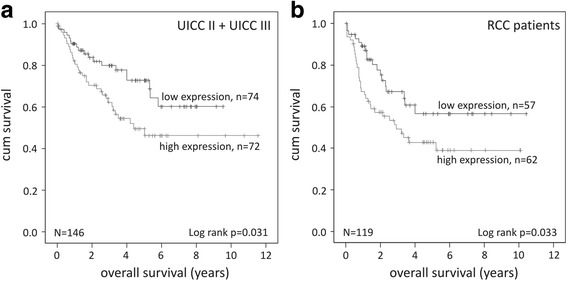
Impact of CK1α RNA expression on prognosis of different subgroups of CRC patients. **a** Kaplan-Meier plot displaying the overall survival of UICC II and UICC III tumors of CRC patients, divided according to relative CK1α RNA expression. **b** Kaplan-Meier plot displaying the overall survival of RCC patients, divided according to relative CK1α RNA expression. CK1α RNA expression in colorectal tumor tissue of all grades was relatively quantified by qPCR using specific primers. HPRT gene served as reference gene. Graphs were created using IBM SPSS Statistics 20

Summarizing the subgroups of patients assigned to UICC II and UICC III stage revealed significantly decreased overall survival rates when CK1α RNA expression levels are elevated (*p* = 0.031; Fig. 3A). Subdividing patients according to tumor localization in right colon cancer (RCC) and left colon cancer (LCC) patients exhibited significantly increased survival rates in RCC patients with tumors expressing low CK1α RNA levels (*p* = 0.033; Fig. 3B). In contrast, no significant correlation between overall survival rates and CK1α expression levels could be observed in LCC patients (*p* = 0.470; Additional file 6). Within this subgroup of tumor localization no significant differences in median CK1α expression occurred (*p* = 0.523; Table 2).

### Correlations between CK1α RNA expression and survival analysis

The results of Cox univariate and multivariate analyses (UVA and MVA, respectively) (Table 3) revealed that low survival rates significantly correlate with high CK1α RNA expression levels (UVA *p* = 0.045, hazard ratio HR = 1.46 and MVA *p* = 0.010, HR = 1.63) and UICC stage (UVA *p* < 10^6^, HR = 1.70 and MVA p < 10^− 6^, HR = 1.78). Furthermore, female sex (UVA *p* = 0.037, HR = 0.82 and MVA *p* = 0.025, HR = 0.65) significantly decreases risk of CRC related death. Additionally, older age (UVA *p* = 0.002, HR = 1.02 and MVA p < 10^− 5^, HR = 1.03) is a significant predictor of a low overall survival rate. Other variables (anamnestic raised indications for smoking or excessive alcohol consumption, tumor localization, occurrence of relapses and treatment with chemotherapy) did not contribute significantly according to forward and backward stepwise likelihood-ratio procedures for model comparison (see supplemental Fig. 2).

**Table 3:** Multivariate and univariate analysis of survival rate in CRC

	Multivariate			Univariate		
Variable	HR	95% CI	*p* value	HR	95% CI	*p* value
CK1α expression						
Low	1.00	1.123–2.351	0.010*	1.00	1.008–2.105	0.045*
High	1.63			1.46		
UICC Stage						
I < II < III < IV	1.78	1.480–2.145	< 10^−6^***	1.70	1.417–2.046	< 10^−6^***
Gender						
Male	1.00	0.442–0.946	0.025*	1.00	0.679–0.988	0.037*
Female	0.65			0.82		
Age						
Ratio scale	1.03	1.016–1.050	< 10^−3^*	1.02	1.009–1.040	0.002**

*p* value was determined using Cox proportional hazards model. * p < 0.05; **p < 0.01; ***p < 0.001

*Abbreviations: HR* hazard ratio*, 95% CI* 95% confidence interval

## Discussion

Colorectal cancer (CRC) is the fourth most common cancer globally and the third leading cause of cancer related deaths [1]. An increasing age can elevate the probability of developing CRC as well as polyps, inflammatory bowel diseases, and hereditary factors in the patients’ anamnesis. The best chance for cure is provided by complete surgical resection of the tumor. However, despite the fact that novel diagnostic tools as well as improved treatment strategies have been emerged in the past years, prognosis of CRC patients with advanced and metastatic tumor stage still remains poor with an average survival of less than 30 months [21]. Consequently, novel targeted therapeutic approaches are urgently needed. Therefore, it is essential to understand molecular mechanisms of CRC. Uncovering disease associated pathways as well as their regulation and interaction, and identifying prognostic biomarkers might provide starting points for targeted therapy with increased selectivity, efficiency and reduced toxicity. Research in CRC reveals potential target molecules including members of the CK1 family. CK1 family members have been described to influence the activity of key regulatory proteins and signal integration molecules including β-catenin, p53, and MDM2, thereby regulating Wnt-signaling, cell cycle progression, and apoptosis induction. Importantly, all these pathways are well known for their role in tumor development and progression. Accordingly, deregulation of CK1 expression and/or activity is directly linked to tumor development and progression and has already been reported in various tumor entities, highlighting CK1 isoforms as attractive targets in tumor therapy (reviewed in [15]).

Microarray data analyses from different cancer cell lines using CellMiner™ database indicate high-level expression of CK1α in various colon and rectum cancer cell lines [20]. However, so far, the role of CK1α as a prognostic marker in CRC has not been reported. In this study, we investigated CK1α RNA expression in CRC as a potential prognostic biomarker by comparing RNA expression levels in colorectal tumor tissue and healthy bowel tissue. Our results revealed that CK1α expression levels are significantly increased in tumor tissue compared to normal tissue, consequently indicating that CK1α is involved in developing and/or proceeding malignant characteristics of tumor cells. Since we found low expression levels in surviving individuals, it might be hypothesized that increased CK1α expression levels correlate with poor prognosis of CRC patients. Survival analyses, including Kaplan-Meier estimations and Cox regression analyses revealed that high CK1α expression levels in tumors are significantly associated with poor overall survival rates of CRC patients, indicating that CK1α is an independent negative prognostic factor in CRC. Interestingly, CK1α expression levels appear to vary in different cancer entities. Therefore, it remains to be shown whether our finding may be transferable to other cancers and if so, to which cancers. For example melanoma cells or lung cancer cells possess low-level expression [14]. Additionally, despite availability of comparable survival information a link between prognosis and CK1α expression levels cannot be transferred to every tumor entity. A significant influence of CK1α expression levels on survival rates is demonstrated amongst others in breast cancer, leukemia, multiple myeloma, lung cancer, and diffuse large B cell lymphoma. For instance, in diffuse large B cell lymphoma a prolonged survival is connected with low CK1α expression levels, whereas in lung cancer high-level expression of CK1α is linked to better outcome [14]. This indicates that CK1α expression levels cannot be used as a general prognostic marker and have to be determined individually in every tumor entity. Our study identifies that CK1α expression influences the overall survival of colorectal cancer patients since patients with short survival times show a statistically significant higher CK1α expression. Thus, we have identified CK1α expression as potential diagnostic or therapeutic applicability for colorectal cancer. However, our data do not provide evidence that CK1α expression may influence the outcome of a certain therapy and can therefore not be used as a predictive marker.

We previously reported a prognostic relevance of CK1δ expression in CRC patients, especially for those with low-grade tumors (Grade 1, Grade 2) [17]. In the present study we could show that high CK1α expression levels strongly correlate with poor survival in high-grade (Grade 3, Grade 4) CRC patients, indicating that CK1α might be used as a biomarker in poorly differentiated cancers, whereas CK1δ represents a prognostic marker in highly differentiated CRC.

Interestingly, we found lower levels of both CK1 isoforms in surviving patients, indicating an involvement of CK1α and CK1δ in malignancy-associated pathways resulting in poor prognosis. Since the APC/Wnt/β-catenin pathway is known to play a major role in colorectal carcinogenesis and since both CK1 isoforms play regulatory roles in Wnt signaling it can be speculated that overexpression of CK1α and δ leads to a Wnt/β-catenin-dependent malignant phenotype of colorectal tumor cells [15, 22, 23]. However, this hypothesis has to be investigated in future experiments unraveling the molecular mechanisms behind CK1α/δ related colorectal carcinogenesis.

Since increased expression levels are associated with poor survival rates, CK1α might represent an attractive drug target in new CRC therapy concepts. Intriguingly, the prognostic relevance of high CK1α expression was explicitly high in UICC II and UICC III stage CRC. This patient cohort usually undergoes surgical resection with a curative intent. However, these patients exhibit a high risk of tumor recurrence due to large primary tumors and/or lymph node metastases at time of diagnosis. Therefore, these patients usually received adjuvant treatment upon surgical resection to decrease risk of recurrence and prolong overall survival. Interestingly, within the cohort patients with poorer survival show higher CK1α expression. Consequently, patients with high CK1α expression are less likely to benefit from conventional adjuvant chemotherapy to prevent recurrence providing the chance for cure. Moreover, based on our findings it is important to characterize the effects of CK1α downregulation by CK1α specific inhibitors or biological tools to receive information whether these patients would benefit from a CK1α targeting therapy in regard to long-term survival, in particular in chemoresistant cancers. Recently, D4476 has been described as a potent inhibitor of CK1α [24–26]**,** although previously having been described as CK1δ/ε specific inhibitor [27, 28]. Furthermore, the effects of the anticancer drug lenalidomide are partially due to the initiation of proteasomal degradation of CK1α [29, 30]. Nevertheless, additional pre-clinical and clinical testing of CK1α inhibitors or agents inducing CK1α degradation or inhibiting the interaction of CK1α with cellular proteins [31] are urgently needed to further investigate their therapeutic potential.

In accordance with the published data a tumor localization-dependent correlation between survival and kinase expression was detected [17]. High-level CK1α expressing tumors of the right colon (right colon cancer, RCC) correlate with poor outcome of the patients, whereas overall survival is not affected by CK1α expression of left-sided tumors (left colon cancer, LCC). Several reports distinguish right and left colon by differences in their biological properties. It has been demonstrated that embryologic origin, vascular supply, as well as composition and density of immune cells and microbiota differ in right and in left colon [32]. Furthermore, genes involved in tumorigenesis-associated signaling pathways, cell cycle, proliferation, cell death, stress response, DNA replication, and damage repair have been reported to be differentially expressed, consequently leading to different oncogenic patterns [33]. According to Vogelstein’s adenoma-carcinoma sequence a stepwise pattern of mutational inactivation of tumor suppressors and activation of oncogenes initiates and proceeds to colorectal carcinogenesis [5]. So far, the role of CK1 isoforms within this sequence is unknown but several reports suggest a role of CK1 in regulating p53 activity, which in turn is inactivated in the majority of high-grade colorectal cancers [15, 34]. Furthermore, differences in the microenvironment, especially the release of pro-inflammatory cytokines, mainly released by immune cells (e.g. neutrophils and M1 macrophages), pro-inflammatory adipocytokines (e.g. leptin, visfatin) and fatty acids secreted by adipocytes accelerate adenoma carcinoma transition and can significantly promote migration and invasion of CRC cells via induction of epithelial mesenchymal transition [35–38]. In this respect, it can be speculated that CK1α expression might be involved in regulating microenvironment and oncogenic pathways in particular important in right-sided colon carcinogenesis, especially against the background that CK1α expression is similar in LCC and RCC. In order to investigate the impact of CK1α on RCC tumorigenesis further studies are crucial.

## Conclusion

In summary, we could show that CK1α RNA overexpression in colorectal tumor tissue significantly correlates with poor outcome. Especially in patients with poorly differentiated tumors CK1α might have prognostic relevance. Interestingly, UICC II and UICC III patients, a subset of patients that usually receive adjuvant chemotherapy upon surgical resection, did also show impaired survival when CK1α was elevated. Therefore, these patients might benefit from combining adjuvant chemotherapy with a CK1α inhibitor.

## Additional files

Additional file 1: Raw data of analyzed patients. Table summarizing the patients’ raw data used for SPSS analysis. Patients have been anonymized and their birth date, surgery date etcetera excluded. Data to comprehend analysis (patients’ age, patients’ sex, observation period, relapse, chemotherapy post-surgery, tumor localization, grading, UICC stage, relative CK1α expression) are listed. (XLSX 53 kb)

Additional file 2: Individual hazards of the CK1α expression level at different cutoff values. The Cox-regression model was fitted with different cutoff values for the categorization of low vs. high relative CK1α expression. The indicator variable was coded as 1, if the expression value was larger than the cutoff value. Additional covariates were UICC, age and sex. There is an increase of the CK1α-related hazard up to expression values of 1.6, the individual hazard is significant for cutoff levels between 2.5 and 3.1 (lower limit of the 95% confidence band (dashed lines) > 1). (TIFF 95 kb)

Additional file 3: Selection of covariates with significant independent hazard for the Cox-regression model. Tables A) and B) display fit results of a stepwise inclusion of variables according to the forward-LR (forward-*likelihood ratio*) procedure of SPSS. The backward-LR procedure ends with the same model after 6 steps. Covariates under consideration were the UICC score (UICC), patient gender (sex) and age (age), a categorical variable for the cutoff of CK1α expression (CK1alpha_Low_High_cutoff at relative CK1α expression > 2.6) and indicator variables for post-surgery chemotherapy (post_OP_chemo), tumor location left, right colon or transversum (Left_Right_Colon), tumor recurrence (recurrence), smoking (smoker) and alcohol consumption (alcohol). For each subsequent step the procedure included the variable with the lowest *p*-value (Sig. in Table B) when added to the model at the current step (Table A). The procedure stopped the inclusion process at step 4 because no p-value was smaller than 0.05. (TIFF 50 kb)

Additional file 4: Gender-specific impact of CK1α RNA expression on prognosis of CRC patients. Kaplan-Meier plot displaying the overall survival of female and male CRC patients, divided according to relative CK1α RNA expression. CK1α RNA expression in colorectal tumor tissue of male and female CRC patients was relatively quantified by qPCR using specific primers. HPRT gene served as reference gene. Graphs were created using IBM SPSS Statistics 20. (TIFF 46 kb)

Additional file 5: Impact of CK1α RNA expression on prognosis of low-grade CRC patients. Kaplan-Meier plot displaying the overall survival of Grade 1 and Grade 2 tumors of CRC patients, divided according to relative CK1α RNA expression. CK1α RNA expression in colorectal tumor tissue of Grade 1 and Grade 2 tumors was relatively quantified by qPCR using specific primers. HPRT gene served as reference gene. Graphs were created using IBM SPSS Statistics 20. (XPDF 50 kb)

Additional file 6: Impact of CK1α RNA expression on prognosis of LCC patients. Kaplan-Meier plot displaying the overall survival of LCC patients, divided according to relative CK1α RNA expression. CK1α RNA expression in colorectal tumor tissue of LCC patients was relatively quantified by qPCR using specific primers. HPRT gene served as reference gene. Graphs were created using IBM SPSS Statistics 20. (PDF 48 kb)

## Abbreviations

APC: Adenomatous polyposis coli
CK1: Casein kinase 1
CRC: Colorectal cancer
DCC: Deleted in colorectal cancer
HNPCC: *Hereditary Non-Polyposis Colorectal Cancer*
IHC: Immunohistochemistry
LCC: Left colon cancer
MDM2: Mouse double minute 2 homolog
MVA: Multivariate analysis
RCC: Right colon cancer
TP53: Tumor protein 53
UICC: Union for International Cancer Control
UVA: Univariate analysis

## References

[1] Ferlay J, Soerjomataram I, Dikshit R, Eser S, Mathers C, Rebelo M, Parkin DM, Forman D, Bray F (2015). Cancer incidence and mortality worldwide: sources, methods and major patterns in GLOBOCAN 2012. Int J Cancer.

[2] Haggar FA, Boushey RP (2009). Colorectal cancer epidemiology: incidence, mortality, survival, and risk factors. Clin Colon Rectal Surg.

[3] Yu YN, Fang JY (2015). Gut microbiota and colorectal cancer. Gastrointest Tumors.

[4] Bujanda L, Cosme A, Gil I, Arenas-Mirave JI (2010). Malignant colorectal polyps. World J Gastroenterol.

[5] Fearon ER, Vogelstein B (1990). A genetic model for colorectal tumorigenesis. Cell.

[6] Jasperson KW, Tuohy TM, Neklason DW, Burt RW (2010). Hereditary and familial colon cancer. Gastroenterology.

[7] Grady WM (2003). Genetic testing for high-risk colon cancer patients. Gastroenterology.

[8] Boland CR, Goel A (2010). Microsatellite instability in colorectal cancer. Gastroenterology.

[9] Gatalica Z, Vranic S, Xiu J, Swensen J, Reddy S (2016). High microsatellite instability (MSI-H) colorectal carcinoma: a brief review of predictive biomarkers in the era of personalized medicine. Familial Cancer.

[10] Heinemann V, Douillard JY, Ducreux M, Peeters M (2013). Targeted therapy in metastatic colorectal cancer -- an example of personalised medicine in action. Cancer Treat Rev.

[11] Siegel R, Ma J, Zou Z, Jemal A (2014). Cancer statistics, 2014. CA Cancer J Clin.

[12] Longley DB, Johnston PG (2005). Molecular mechanisms of drug resistance. J Pathol.

[13] Hammond WA, Swaika A, Mody K: Pharmacologic resistance in colorectal cancer: a review. Ther Adv Med Oncol 2016, 8(1):57–84. 10.1177/1758834015614530 PMC4699262 26753006

[14] Schittek B, Sinnberg T (2014). Biological functions of casein kinase 1 isoforms and putative roles in tumorigenesis. Mol Cancer.

[15] Knippschild U, Krueger M, Richter J, Xu P, García-Reyes B, Peifer C, Halekotte J, Bakulev V, Bischof J. The CK1 family: contribution to cellular stress response and its role in carcinogenesis. Front Oncol. 2014;4(96) 10.3389/fonc.2014.00096 PMC4032983 24904820

[16] Modak C, Chai J (2009). Potential of casein kinase I in digestive cancer screening. World J Gastrointest Oncol.

[17] Richter J, Rudeck S, Kretz AL, Kramer K, Just S, Henne-Bruns D, Hillenbrand A, Leithauser F, Lemke J, Knippschild U. Decreased CK1delta expression predicts prolonged survival in colorectal cancer patients. Tumour Biol. 2016; 10.1007/s13277-015-4745-8 26738869

[18] Bosmann FT, Carneiro F, Hruban RH, Theise ND: World Health Organization Classification of Tumours, 4th edn. Lyon: International Agency for Research on Cancer (IARC); 2010.

[19] Sobin LH, Gospodarowicz MK, Wittekind C (2011). TNM classification of malignant tumours: John Wiley & Sons.

[20] Shankavaram UT, Varma S, Kane D, Sunshine M, Chary KK, Reinhold WC, Pommier Y, Weinstein JN (2009). CellMiner: a relational database and query tool for the NCI-60 cancer cell lines. BMC Genomics.

[21] Aran V, Victorino AP, Thuler LC, Ferreira CG. Colorectal cancer: epidemiology, disease mechanisms and interventions to reduce onset and mortality. Clin Colorectal Cancer. 2016; 10.1016/j.clcc.2016.02.008 26964802

[22] Bienz M, Clevers H (2000). Linking colorectal cancer to Wnt signaling. Cell.

[23] Fodde R, Smits R, Clevers H (2001). APC, signal transduction and genetic instability in colorectal cancer. Nat Rev Cancer.

[24] Honaker Y, Piwnica-Worms H (2010). Casein kinase 1 functions as both penultimate and ultimate kinase in regulating Cdc25A destruction. Oncogene.

[25] Jaras M, Miller PG, Chu LP, Puram RV, Fink EC, Schneider RK, Al-Shahrour F, Pena P, Breyfogle LJ, Hartwell KA (2014). Csnk1a1 inhibition has p53-dependent therapeutic efficacy in acute myeloid leukemia. J Exp Med.

[26] Lantermann AB, Chen D, McCutcheon K, Hoffmann G, Frias E, Ruddy D, Rakiec D, Korn J, McAllister G, Stegemeier F (2015). Inhibition of casein kinase 1 alpha prevents acquired drug resistance to Erlotinib in EGFR-mutant non-small cell lung cancer. Cancer Res.

[27] Bain J, Plater L, Elliott M, Shpiro N, Hastie CJ, McLauchlan H, Klevernic I, Arthur JS, Alessi DR, Cohen P (2007). The selectivity of protein kinase inhibitors: a further update. Biochem J.

[28] Rena G, Bain J, Elliott M, Cohen P (2004). D4476, a cell-permeant inhibitor of CK1, suppresses the site-specific phosphorylation and nuclear exclusion of FOXO1a. EMBO Rep.

[29] Kronke J, Fink EC, Hollenbach PW, MacBeth KJ, Hurst SN, Udeshi ND, Chamberlain PP, Mani DR, Gandhi AK, Svinkina T (2015). Lenalidomide induces ubiquitination and degradation of CK1α in del(5q) MDS. Nature.

[30] Vo MC, Nguyen-Pham TN, Lee HJ, Jaya Lakshmi T, Yang S, Jung SH, Kim HJ, Lee JJ (2017). Combination therapy with dendritic cells and lenalidomide is an effective approach to enhance antitumor immunity in a mouse colon cancer model. Oncotarget.

[31] Huart AS, MacLaine NJ, Narayan V, Hupp TR (2012). Exploiting the MDM2-CK1alpha protein-protein interface to develop novel biologics that induce UBL-kinase-modification and inhibit cell growth. PLoS One.

[32] Bufill JA (1990). Colorectal cancer: evidence for distinct genetic categories based on proximal or distal tumor location. Ann Intern Med.

[33] Glebov OK, Rodriguez LM, Nakahara K, Jenkins J, Cliatt J, Humbyrd CJ, DeNobile J, Soballe P, Simon R, Wright G *et al*: distinguishing right from left colon by the pattern of gene expression. Cancer Epidemiol Biomark Prev 2003, 12(8):755–762. 12917207

[34] Zlobec I, Lugli A (2008). Prognostic and predictive factors in colorectal cancer. J Clin Pathol.

[35] Zeng H, Ishaq SL, Zhao FQ, Wright AG (2016). Colonic inflammation accompanies an increase of beta-catenin signaling and Lachnospiraceae/Streptococcaceae bacteria in the hind gut of high-fat diet-fed mice. J Nutr Biochem.

[36] Yang J, Zhang K, Song H, Wu M, Li J, Yong Z, Jiang S, Kuang X, Zhang T. Visfatin is involved in promotion of colorectal carcinoma malignancy through an inducing EMT mechanism. Oncotarget. 2016; 10.18632/oncotarget.8615 PMC5078014 27058759

[37] Gong Y, Dou LJ, Liang J (2014). Link between obesity and cancer: role of triglyceride/free fatty acid cycling. Eur Rev Med Pharmacol Sci.

[38] Laiyemo AO (2014). The risk of colonic adenomas and colonic cancer in obesity. Best Pract Res Clin Gastroenterol.

