# Supplementary figures and images for: CK1α overexpression correlates with poor survival in colorectal cancer
Source: BMC Cancer. 2018 Feb 6;18:140. doi: 10.1186/s12885-018-4019-0 (PMC5801892; doi:10.1186/s12885-018-4019-0)

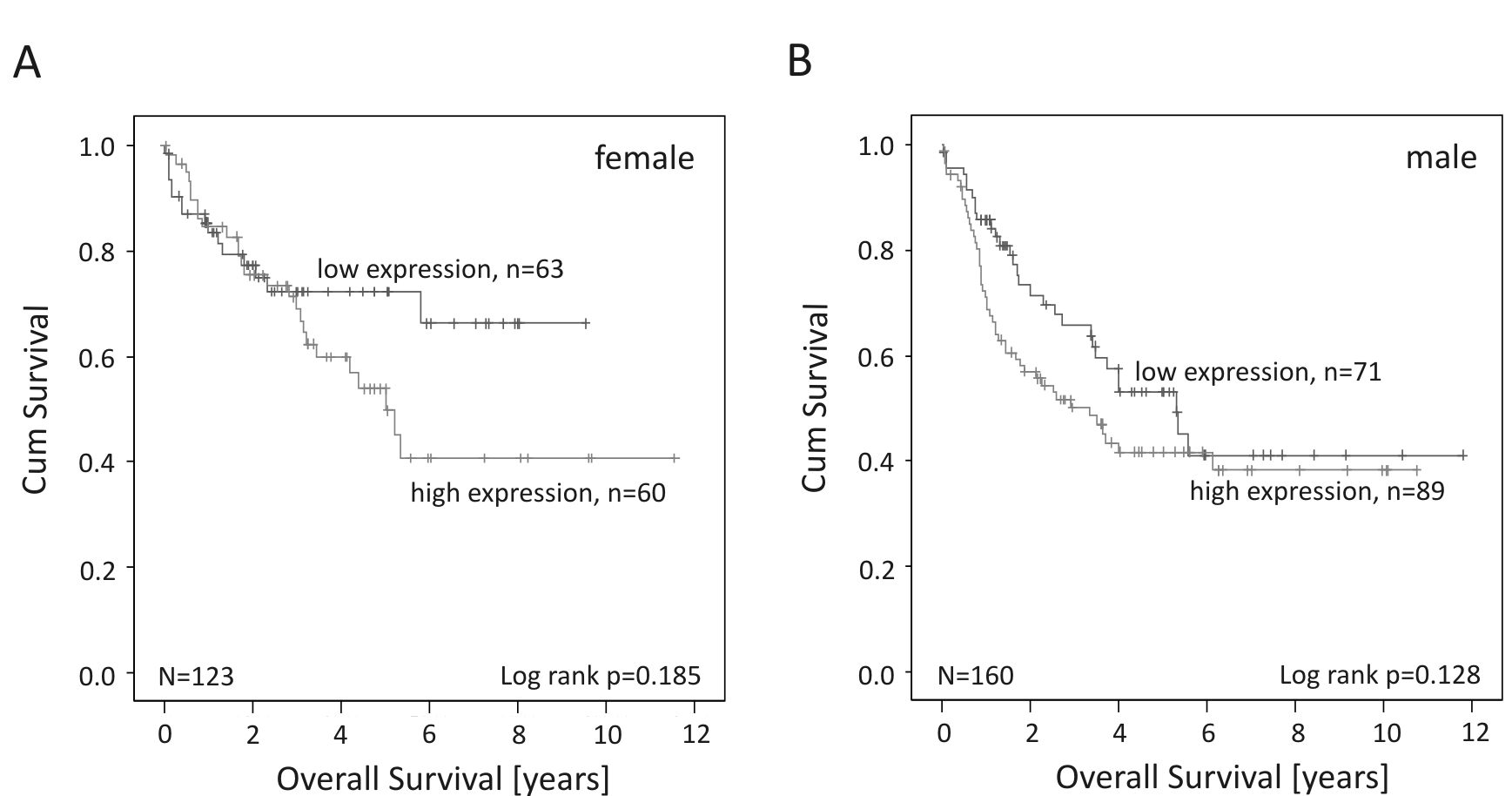

Supplement: Supplementary file 2 — Individual hazards of the CK1α expression level at different cutoff values. The Cox-regression model was fitted with different cutoff values for the categorization of low vs. high relative CK1α expression. The indicator variable was coded as 1, if the expression value was larger than the cutoff value. Additional covariates were UICC, age and sex. There is an increase of the CK1α-related hazard up to expression values of 1.6, the individual hazard is significant for cutoff levels between 2.5 and 3.1 (lower limit of the 95% confidence band (dashed lines) > 1). (TIFF 95 kb) [file 12885_2018_4019_MOESM2_ESM.tif]

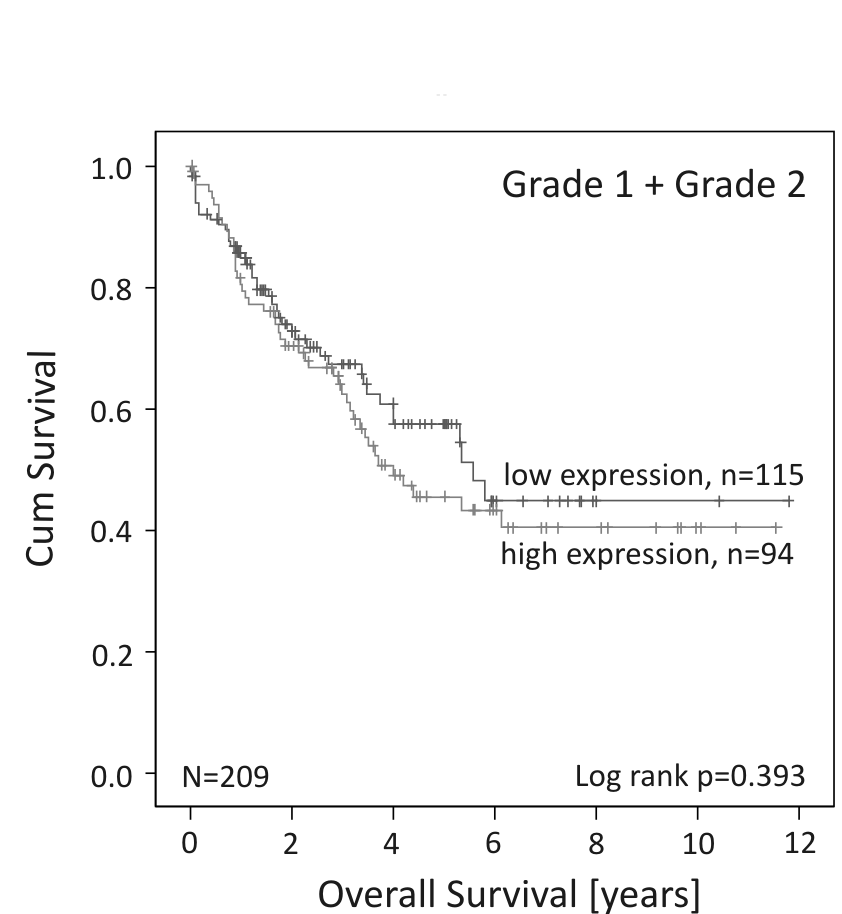

Supplement: Supplementary file 3 — Selection of covariates with significant independent hazard for the Cox-regression model. Tables A) and B) display fit results of a stepwise inclusion of variables according to the forward-LR (forward-likelihood ratio) procedure of SPSS. The backward-LR procedure ends with the same model after 6 steps. Covariates under consideration were the UICC score (UICC), patient gender (sex) and age (age), a categorical variable for the cutoff of CK1α expression (CK1alpha_Low_High_cutoff at relative CK1α expression > 2.6) and indicator variables for post-surgery chemotherapy (post_OP_chemo), tumor location left, right colon or transversum (Left_Right_Colon), tumor recurrence (recurrence), smoking (smoker) and alcohol consumption (alcohol). For each subsequent step the procedure included the variable with the lowest p-value (Sig. in Table B) when added to the model at the current step (Table A). The procedure stopped the inclusion process at step 4 because no p-value was smaller than 0.05. (TIFF 50 kb) [file 12885_2018_4019_MOESM3_ESM.tif]

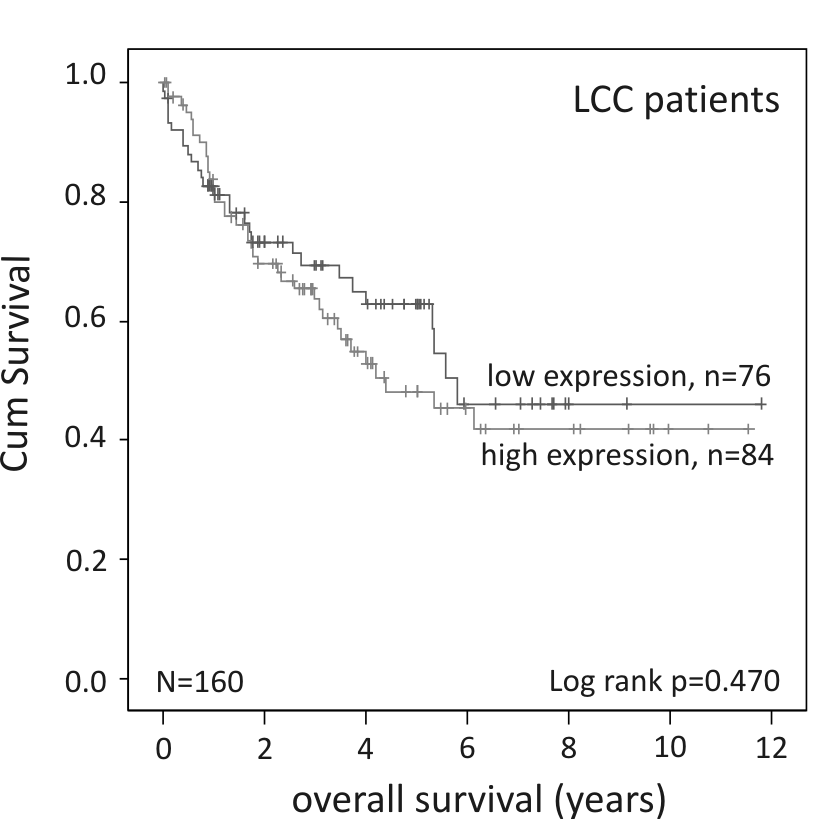

Supplement: Supplementary file 4 — Gender-specific impact of CK1α RNA expression on prognosis of CRC patients. Kaplan-Meier plot displaying the overall survival of female and male CRC patients, divided according to relative CK1α RNA expression. CK1α RNA expression in colorectal tumor tissue of male and female CRC patients was relatively quantified by qPCR using specific primers. HPRT gene served as reference gene. Graphs were created using IBM SPSS Statistics 20. (TIFF 46 kb) [file 12885_2018_4019_MOESM4_ESM.tif]

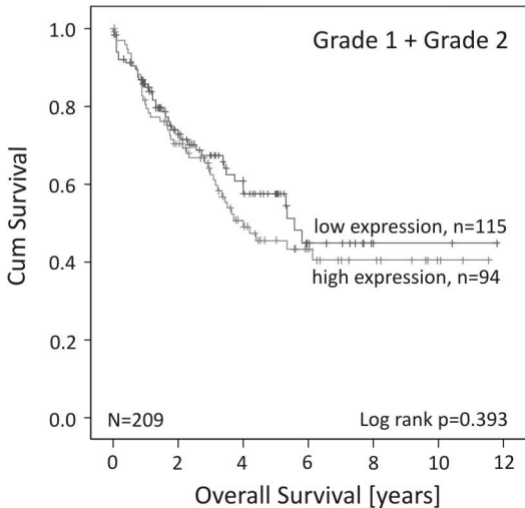

Supplement: Supplementary file 5 — Impact of CK1α RNA expression on prognosis of low-grade CRC patients. Kaplan-Meier plot displaying the overall survival of Grade 1 and Grade 2 tumors of CRC patients, divided according to relative CK1α RNA expression. CK1α RNA expression in colorectal tumor tissue of Grade 1 and Grade 2 tumors was relatively quantified by qPCR using specific primers. HPRT gene served as reference gene. Graphs were created using IBM SPSS Statistics 20. (XPDF 50 kb) [file 12885_2018_4019_MOESM5_ESM.pdf]
