# Supplementary material for: CK1α overexpression correlates with poor survival in colorectal cancer
Source: BMC Cancer. 2018 Feb 6;18:140. doi: 10.1186/s12885-018-4019-0 (PMC5801892; doi:10.1186/s12885-018-4019-0)
Supplement: Supplementary file 6 — Impact of CK1α RNA expression on prognosis of LCC patients. Kaplan-Meier plot displaying the overall survival of LCC patients, divided according to relative CK1α RNA expression. CK1α RNA expression in colorectal tumor tissue of LCC patients was relatively quantified by qPCR using specific primers. HPRT gene served as reference gene. Graphs were created using IBM SPSS Statistics 20. (PDF 48 kb) [file 12885_2018_4019_MOESM6_ESM.pdf]

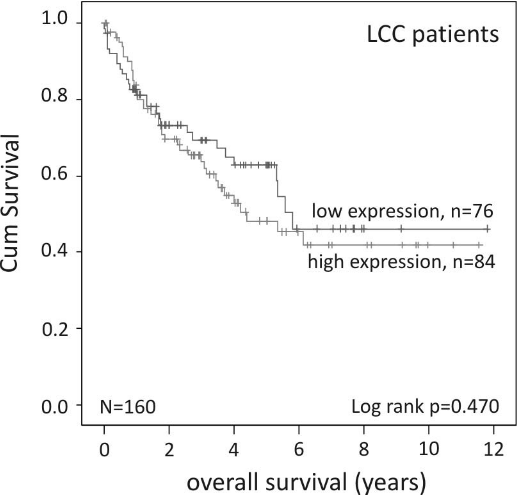
LCC patients
low expression, n=76
high expression, n=84
N=160
Log rank p=0.470
Cum Survival
1.0
0.8
0.6
0.4
0.2
0.0
0
2
4
6
8
10
12
overall survival (years)
